# Development of Local Bleomycin-Induced Hindlimb Model Recapitulating Skin Fibrosis and Vasculopathy of Systemic Sclerosis

**DOI:** 10.1055/a-2908-5229

**Published:** 2026-08-11

**Authors:** Jun Hyeok Kim, Eunchae Shin, Jaechan Koo, Yeon Hee Ryu, Su Jin Lee, Suk-Ho Moon

**Affiliations:** 1 Department of Plastic and Reconstructive Surgery 26713 College of Medicine, The Catholic University of KoreaSeoulKorea (the Republic of); 2 Department of Medical Sciences 37128 College of Medicine, The Catholic University of KoreaSeocho-guSeoulKorea (the Republic of)

**Keywords:** research/experimental, fibrosis, ischemia/reperfusion, methodology, systemic sclerosis, bleomycin, hindlimb ischemia, microvasculopathy, animal model

## Abstract

**Background:**

Systemic sclerosis (SSc) features microvascular injury and progressive fibrosis, often culminating in peripheral ischemia, digital ulcers, and tissue loss. Existing animal models typically capture either fibrosis (e.g., bleomycin [BLM] skin models) or ischemia (surgical hindlimb ligation) but rarely both simultaneously in an extremity.

**Methods:**

We established a non-surgical, localized model by administering subcutaneous BLM (300 μg per day) to four peri-ankle sites of the right hindlimb in BALB/c and C57BL/6 mice for 14 consecutive days, followed by a 7-day rest. Outcomes included clinical scoring (edema, joint stiffness, skin thickening), laser Doppler perfusion imaging (LDPI; ischemic/contralateral perfusion ratio), histology (hematoxylin and eosin; dermal thickness; inflammation; collagen deposition), quantitative RT-PCR (RT-qPCR) for fibrotic and vascular markers, and immunohistochemical (IHC) staining for collagen type I alpha 1 chain (COL1A1), alpha-smooth muscle actin (α-SMA), cluster of differentiation (CD)31, and CD34 with ImageJ-based quantification.

**Results:**

BLM induced persistent hindlimb edema and contracture with significantly reduced perfusion measured by LDPI compared with the contralateral limb, persisting after the rest period. Histology revealed marked dermal thickening with dense collagen and leukocyte infiltration. RT-qPCR demonstrated dose-dependent upregulation of α-SMA and transforming growth factor β (TGF-β) mRNA, and IHC confirmed increased COL1A1- and α-SMA-positive areas alongside reduced CD31/CD34 capillary density in BLM-treated limbs. The protocol was technically simple and showed reproducible results.

**Conclusions:**

Local peri-ankle BLM produces a chronic fibrotic phenotype accompanied by microvascular alteration in an accessible limb, complementing classic SSc models and surgical hindlimb ischemia. This platform enables functional perfusion readouts alongside fibrosis endpoints and is well-suited for preclinical screening of proangiogenic or antifibrotic strategies of interest to reconstructive surgery.

## Introduction

Systemic sclerosis (SSc) is a complex autoimmune connective tissue disorder marked by the triad of microvascular dysfunction, inflammatory activation, and progressive fibrosis of skin and internal organs. 1 2 Clinically, SSc patients often suffer from digital ischemia, recurrent ulcers, and tissue loss, which represent unmet reconstructive challenges. To explore pathophysiology and therapeutic approaches, robust animal models are indispensable. 3 4

The bleomycin (BLM)-induced skin fibrosis model remains one of the most extensively used to mimic SSc-related dermal changes. 5 In this model, repetitive intradermal or subcutaneous injections of BLM result in progressive dermal thickening, immune cell infiltration, increased collagen deposition, and the appearance of alpha-smooth muscle actin (α-SMA)-positive myofibroblasts for skin fibrosis induction. However, this classic BLM-based model is often confined to dorsal skin regions, limiting its translational relevance to limbs, and it typically lacks functional vascular readouts in the extremities, such as perfusion or angiogenesis assays.

In the vascular research domain, murine hindlimb ischemia (HLI) models—most commonly via femoral artery ligation or excision—have served as a standard platform for investigating angiogenesis, collateral formation, and perfusion recovery, and the established protocol is widely documented. 6 Laser Doppler perfusion imaging (LDPI) is a frequent noninvasive method to monitor microvascular flow recovery in such models. Its utility stems from its reproducibility and its ability to compare perfusion across time points in the same animal. 7

Nevertheless, the majority of HLI models lack the fibrotic soft tissue milieu characteristic of SSc skin, and BLM models lack perfusion dynamics. 8 9 This disconnect limits translational applicability for ischemic fibrotic tissues. Additionally, in peripheral arterial disease research, the authors recommend combining multiple endpoints (perfusion, histology, functional scores) for comprehensive evaluation.

Given these gaps, we hypothesized that local peri-ankle BLM injections could generate a composite environment in a limb encompassing microvascular injury and fibrosis, permitting within-animal perfusion tracking and morphological assessment. We, therefore, developed and characterized a non-surgical, localized hindlimb model incorporating fibrotic and microvascular features.

## Methods

### Animals and Grouping

Eight- to 10-week-old BALB/c mice (used for all quantitative analyses—LDPI, quantitative RT-PCR (RT-qPCR), histology, and immunohistochemical (IHC)—with *n* = 10 per group for dose optimization and reproducibility assessment and *n* = 5 per group for molecular, histologic, and IHC analyses) and C57BL/6 mice (used for initial exploratory dose-ranging only; average body weight ≈20 g) were used. Animals were maintained in a controlled environment (24 ± 2 °C, 50 ± 5% humidity, 12-hour light/dark cycle) with free access to food and water. All procedures conformed to the guidelines of the Institutional Animal Care and Use Committee (approval number CUMC-2020-0347-06).

### Bleomycin Injection Protocol

**Fig. 1:**
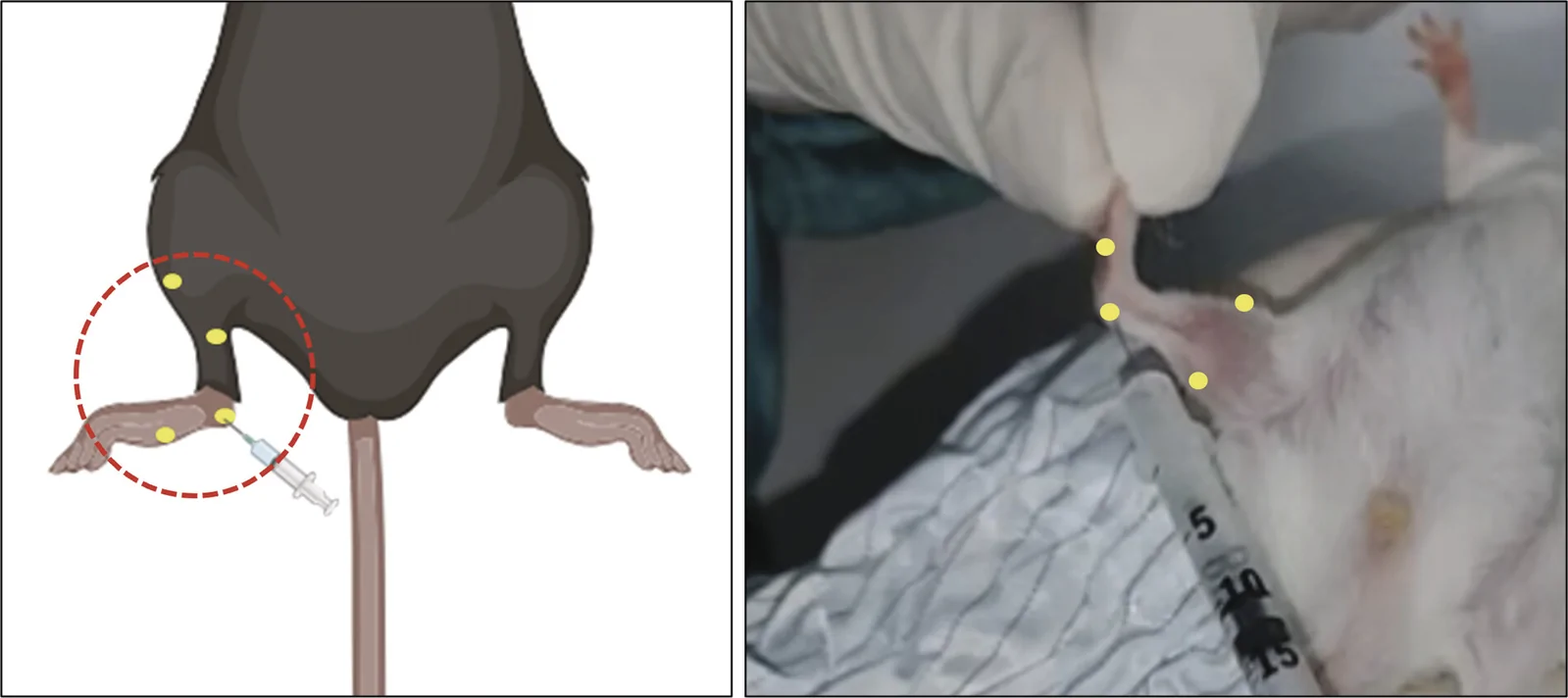
Experimental design and injection sites: Schematic showing subcutaneous bleomycin (300 μg/day) injections at four peri-ankle sites for 14 days followed by a 7-day rest (yellow dots indicate injection points).

BLM sulfate (USP grade; Merck KGaA, Darmstadt, Germany) was freshly prepared in sterile normal saline immediately before injection. The stock solution was adjusted so that each animal received the intended total dose in a constant volume of approximately 50 μL per session. Under light isoflurane anesthesia (1.5–2%), BLM was injected subcutaneously at four peri-ankle sites around the right hindlimb, as illustrated in Fig. 1 . Doses of 200, 250, 300, and 400 μg per mouse were initially tested for dose optimization ( *n* = 10 per dose group). Each dose was administered once every 24 hours for 14 days, followed by a 7-day injection-free rest period. The contralateral limb was left untreated and served as an internal control. The 300 μg dose produced reproducible ischemic and fibrotic phenotypes with minimal mortality ( Supplementary Fig. 1 [available in the online version only]), whereas the 400 μg dose caused approximately 90% lethality (9/10 mice) and was excluded from subsequent experiments.

### Clinical Evaluation

At baseline, day 7, day 14, and day 21, hindlimb photographs were obtained under standardized conditions. Each limb was evaluated for edema, skin induration, and joint (ankle) flexibility/contracture. Animal body weights were monitored regularly. Mice exceeding 15% weight loss or developing ulceration were excluded per humane endpoints.

### Laser Doppler Perfusion Imaging

On day 21, mice were anesthetized and placed supine on a warming pad at approximately 37 °C. Fur on both hindlimbs was removed 24 hours prior to imaging. Perfusion scans were acquired in standardized regions of interest (ROIs; ankle/plantar region). The ischemic ratio was defined as perfusion_right/perfusion_left. LDPI output is reported in perfusion units (PU), and the contralateral limb serves as internal normalization. 10

### Histopathology

At day 21, mice were euthanized, and approximately 1 × 1 cm samples of skin and subcutaneous tissue around the right ankle were excised. Tissues were stained with hematoxylin and eosin (H&E) for morphology and inflammation, and Masson's trichrome for collagen deposition. Vascular remodeling was qualitatively evaluated by assessing lumen narrowing and wall thickening in H&E sections. For histologic quantification, at least three to five non-consecutive sections were analyzed per specimen. Dermal thickness was measured in five or more randomly selected fields per section, and mean values were calculated to minimize sampling bias. Collagen-positive area was quantified from Masson's trichrome-stained sections as the percentage of blue-stained area within the dermis ROI, using ImageJ with auto-thresholding at ×40 magnification.

### Quantitative RT-PCR

Total RNA was extracted from harvested skin tissues using TRIzol Reagent (Invitrogen, Carlsbad, CA, United States) according to the manufacturer's protocol. Briefly, 1 mL of TRIzol was added to each sample, followed by the addition of 200 μL of chloroform. After vigorous mixing and centrifugation at 14,000 rpm and 4 °C for 15 minutes, the aqueous phase was transferred to a fresh tube, and RNA was precipitated with isopropanol, washed with 75% ethanol, and dissolved in 30 μL of RNase-free water. RNA concentration and purity were quantified using a NanoDrop spectrophotometer.

For cDNA synthesis, 1 μg of total RNA was annealed with 2 μL of oligo-dT primers at 70 °C for 10 minutes and then reverse-transcribed in a 20-μL reaction containing 1× first-strand buffer, 0.1 M dithiothreitol, 10 mM deoxynucleoside triphosphate mix, RNase inhibitor, and Moloney murine leukemia virus Reverse Transcriptase (Invitrogen). The reaction was incubated sequentially at 37 °C for 60 minutes and 75 °C for 15 minutes.

Quantitative real-time PCR was performed using a SYBR Green Master Mix (Thermo Fisher Scientific, Waltham, MA, United States) on a QuantStudio 3 Real-Time PCR System (Applied Biosystems). Relative gene expression was normalized to glyceraldehyde-3-phosphate dehydrogenase as an internal reference, and quantification was calculated by the 2 ^−ΔΔ *C* t^ method. Primer sequences used for all target genes are listed in Table 1 .

**Table 1:** Primer sequences used for quantitative RT-PCR

Target gene	Forward primer (5′→3′)	Reverse primer (5′→3′)
*CD34*	GGTGCCAAGCATTTCGGTTT	CAGGAGAAAGGCTGGGTGAAG
*VEGF*	GCTACTGCCGTCCGATTGA	CGCTTTCGTTTTTGACCCTT
*α-SMA*	CCTGACGGGCAGGTGATC	ATGAAAGATGGCTGGAAGAGAGTCT
*TGF-β*	GCCTGAGTGGCTGTCTTTTGA	CACAAGAGCAGTGAGCGCTGAA
*GAPDH*	TGATGACATCAAGAAGGTGGTGAAG	TCCTTGGAGGCCATGTAGGCCAT

Abbreviation: TGF-β, transforming growth factor β; GAPDH, glyceraldehyde-3-phosphate dehydrogenase.

### Immunohistochemistry

IHC staining was performed on formalin-fixed, paraffin-embedded tissue sections. The sections were deparaffinized and rehydrated, followed by heat-induced epitope retrieval in 10 mM sodium citrate buffer (pH 6.0) at 95 to 100 °C for 15 to 20 minutes. Sections were then incubated overnight at 4 °C with the following primary antibodies: anti-collagen type I alpha 1 chain (COL1A1) (1:100; #72026, Cell Signaling Technology), anti-α-SMA (1:100; ab5694, Abcam), anti-cluster of differentiation (CD)34 (1:100; ab81289, Abcam), and anti-CD31 (1:100; 550274, BD Pharmingen). After washing, sections were incubated with horseradish peroxidase (HRP)-conjugated anti-rabbit IgG or HRP-conjugated anti-rat IgG, depending on the host species of each primary antibody. Immunoreactivity was visualized using a 3,3′-diaminobenzidine (DAB) chromogen system, and sections were counterstained with hematoxylin and examined under a light microscope.

For quantification, ImageJ software was used. The ROI was restricted to the dermis. At least three to five non-consecutive sections per specimen were analyzed, with five or more randomly selected fields per section at ×40 magnification. Positive areas for COL1A1 and α-SMA were expressed as a percentage of the total ROI area. Capillary density for CD31 and CD34 was determined by counting the number of positively stained microvessels per unit area. Auto-thresholding was applied consistently across all specimens.

### Statistical Analysis

Perfusion values obtained from LDPI (in PU) were expressed as mean ± standard deviation (SD) and compared within animals using paired *t* -tests. Gene expression data and IHC quantification values were similarly expressed as mean ± SD; differences among dose groups were assessed by one-way analysis of variance (ANOVA) followed by Tukey's post hoc test where appropriate. Statistical significance was defined as *p* < 0.05.

## Results

### Dose Optimization and Clinical Findings

All mice tolerated injections without immediate adverse reactions. Doses of 200 and 250 μg failed to induce consistent ischemic features even after 4 weeks of observation, while higher concentrations produced dose-dependent changes. Mice receiving 300 or 400 μg BLM exhibited marked limb swelling, joint stiffness, and skin thickening by 2 weeks. However, the 400 μg group showed approximately 90% mortality within 2 weeks (9/10 mice), whereas 300 μg yielded reproducible ischemic phenotypes without mortality. Thus, 300 μg was selected as the optimal dose for further analysis.

**Fig. 2:**
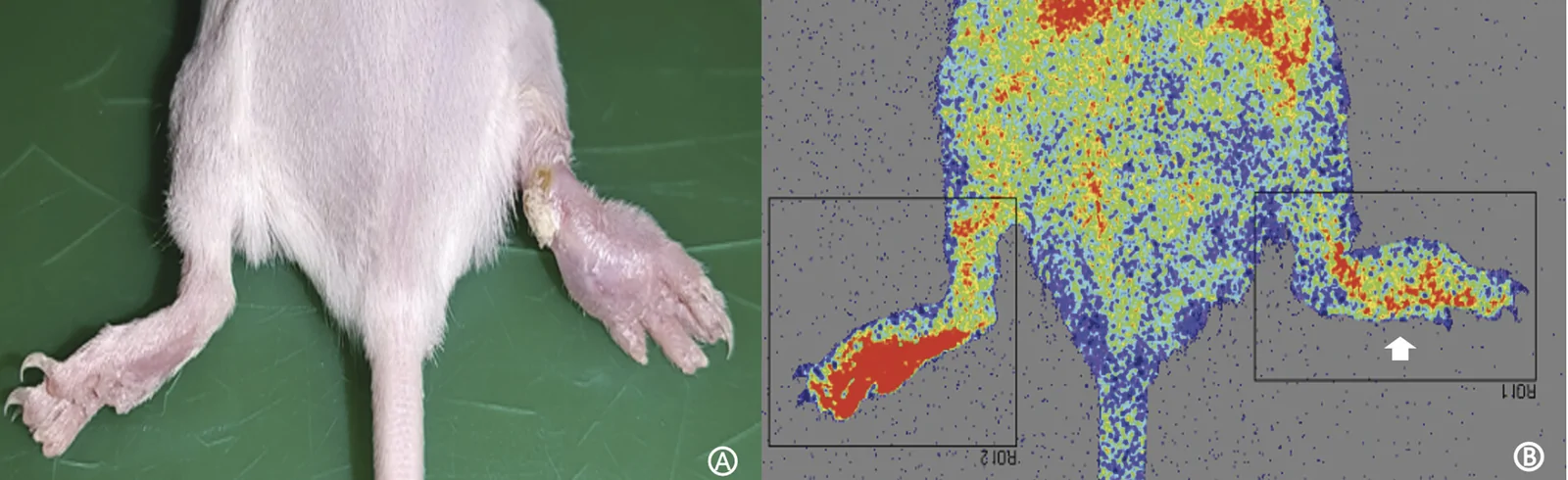
Dose optimization and perfusion assessment. ( **A** ) Localized hindlimb fibrosis with microvascular alteration was reproducibly induced with 300 μg of bleomycin, whereas lower doses caused minimal change. ( **B** ) Laser Doppler imaging at day 21 demonstrating reduced perfusion in the treated limb.

At week 1, mild erythema and edema appeared around the ankle. By weeks 2 to 3, 10/10 mice showed obvious edema and restricted joint mobility ( Fig. 2 ). Skin surface appeared taut and thickened, with mild ulceration in one case but no auto-amputation.

### Perfusion Analysis

**Fig. 3:**
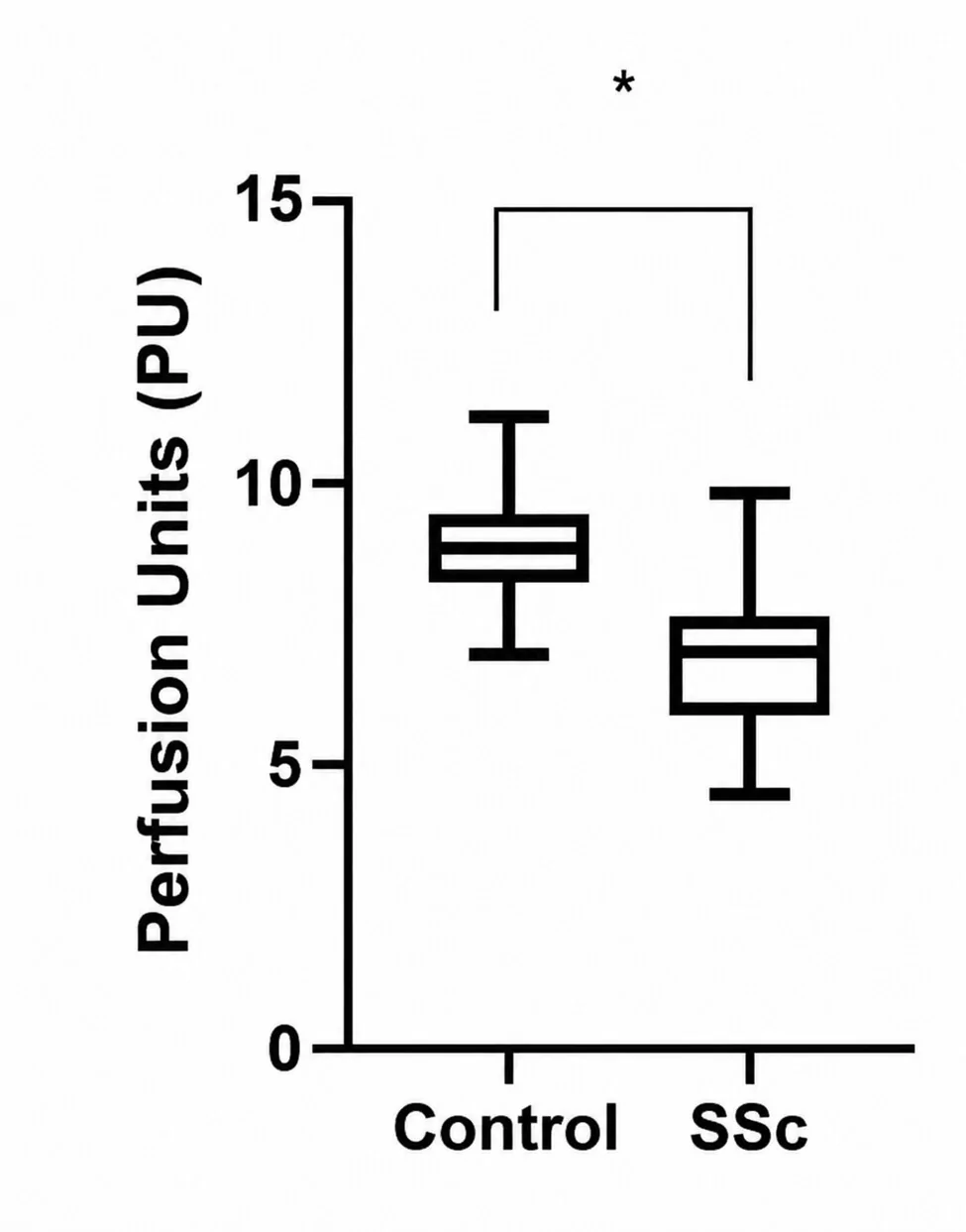
Laser Doppler perfusion analysis: Color-coded LDPI maps reveal reduced perfusion in treated limbs. Quantification shows significantly lower mean perfusion in BLM-treated limbs (6.84 ± 1.37 PU vs. 8.85 ± 0.77 PU; *p* = 0.020). BLM, bleomycin; LDPI, laser Doppler perfusion imaging; PU, perfusion unit; SSc, systemic sclerosis.

LDPI demonstrated markedly reduced perfusion in the BLM-treated hindlimb compared with the contralateral control ( Fig. 2 ). Quantitative analysis showed that mean perfusion was significantly lower in the treated limb (6.84 ± 1.37 PU) than in the control limb (8.85 ± 0.77 PU; paired *t* -test, p = 0.020; Fig. 3 ). Color-coded perfusion maps revealed pronounced hypoperfusion—visible as blue-to-green signals—confined to the right hindlimb, corresponding to the area of edema and contracture observed clinically. Quantitative statistical analysis was conducted at the final time point (day 21), whereas earlier LDPI measurements were used qualitatively to monitor perfusion trends.

### Reproducibility

Ten mice injected with 300 μg BLM exhibited a uniform ischemic phenotype across replicates, confirming high reproducibility of the model.

### Molecular Analysis

**Fig. 4:**
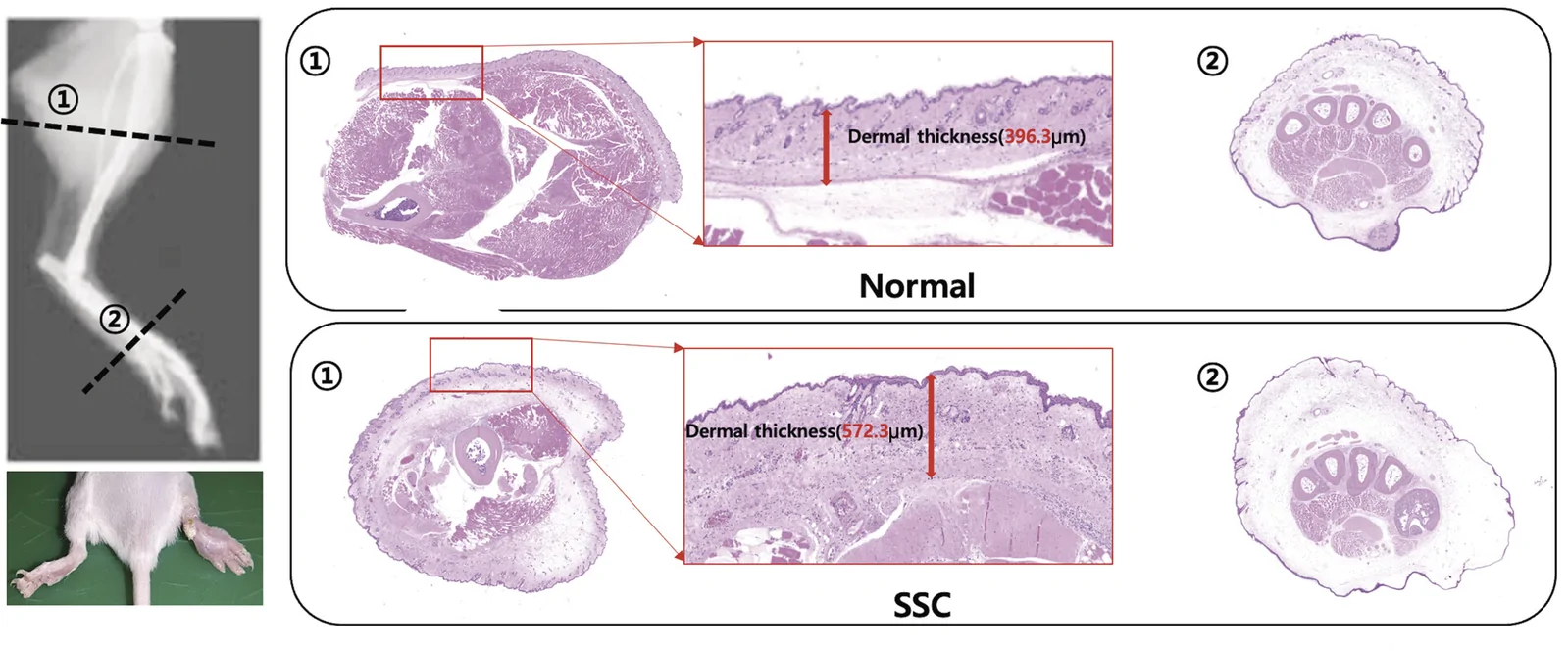
Histopathologic findings: H&E and trichrome staining show thickened dermis (∼570 vs. ∼400 μm), dense collagen, and inflammatory infiltration in BLM-treated skin. BLM, bleomycin; H&E, hematoxylin and eosin.

To further characterize molecular changes associated with fibrosis and vascular alteration, RT-qPCR analysis was performed. Expression of fibrotic markers α-SMA and TGF-β increased following BLM treatment, while angiogenesis-related genes *CD34* and *VEGF* showed dose-dependent alterations compared with controls ( Fig. 4A ).

### Histopathology

**Fig. 5:**
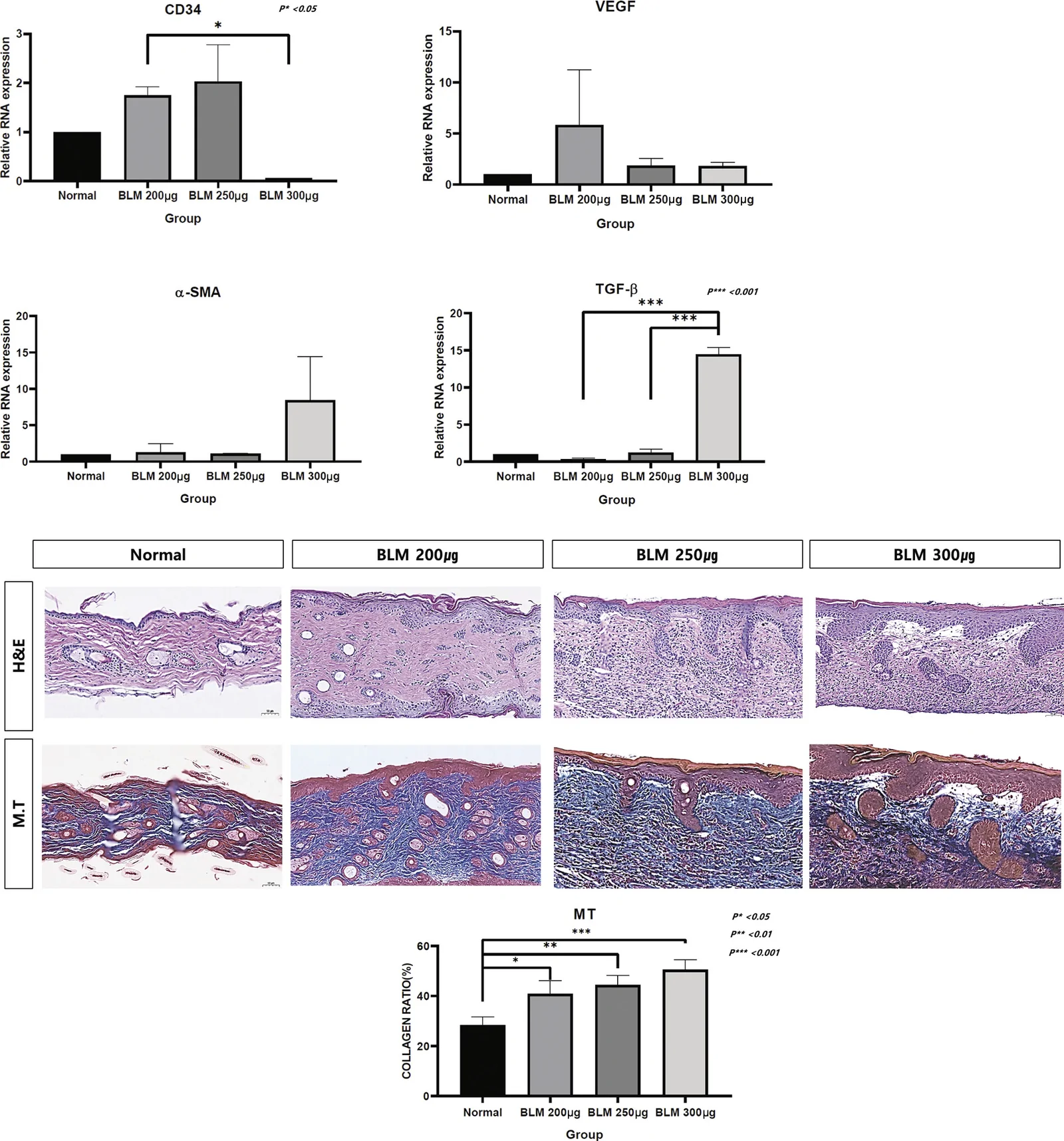
Molecular and histological characterization of fibrosis in bleomycin-treated hindlimb skin. ( **A** ) RT-qPCR analysis of fibrosis- and vascular-related gene expression. Relative mRNA expression levels of CD34, VEGF, α-SMA, and TGF-β were measured in normal controls and bleomycin-treated groups (200, 250, and 300 μg). ( **B** ) Representative hematoxylin and eosin (H&E) and Masson's trichrome staining demonstrate dose-dependent dermal thickening and collagen deposition, with quantitative analysis of collagen-positive area. Data are presented as mean ± SD. Statistical significance is indicated as shown. RT-qPCR, quantitative RT-PCR; SD, standard deviation.

**Fig. 6:**
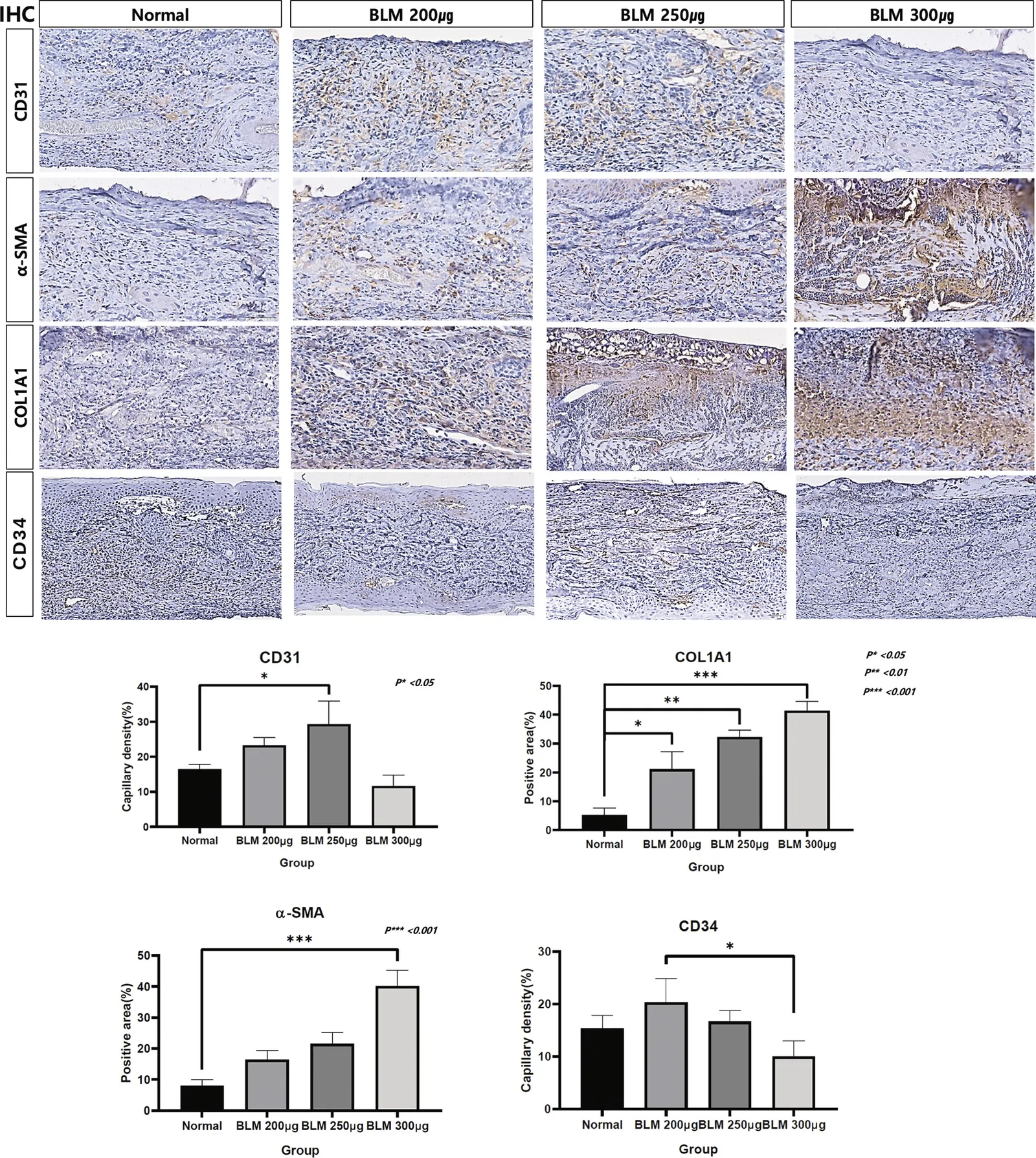
Immunohistochemical analysis of fibrosis and microvascular markers in bleomycin-treated hindlimb skin. ( **A** ) Immunohistochemical (IHC) staining for CD31, α-SMA, COL1A1, and CD34. α-SMA and COL1A1 expression increased in a dose-dependent manner, indicating myofibroblast activation and extracellular matrix accumulation, whereas CD31 and CD34 signals were reduced at the 300 μg dose, indicating decreased microvascular density. ( **B** ) ImageJ-based quantification of positive area (COL1A1, α-SMA) and capillary density (CD31, CD34). Data are presented as mean ± SD. Statistical significance is indicated as shown. SD, standard deviation.

At day 21, histological sections from BLM-treated hindlimbs displayed extensive dermal thickening, inflammatory infiltration, and microvascular alteration ( Fig. 5 ). Histological examination revealed a markedly thickened dermis in BLM-treated mice (approximately 570 μm) compared with controls (approximately 400 μm), accompanied by dense collagen deposition and inflammatory infiltration. These changes were confirmed by H&E and Masson's trichrome staining, with quantitative analysis demonstrating increased collagen-positive area at higher BLM doses ( Fig. 4B ). Enlarged myonuclei in panniculus carnosus and perivascular leukocyte clusters were observed, indicating chronic inflammation and fibrotic vascular remodeling. IHC staining combined with ImageJ-based quantification demonstrated increased COL1A1- and α-SMA-positive areas in BLM-treated tissue, indicating extracellular matrix accumulation and myofibroblast activation ( Fig. 6A ). To assess microvascular changes, IHC staining for CD31 and CD34 was performed. BLM-treated hindlimbs, particularly at the 300 μg dose, exhibited reduced microvascular marker expression, and ImageJ-based quantitative analysis confirmed decreased capillary density compared with controls ( Fig. 6B ).

## Discussion

In this work, we introduce a localized peri-ankle BLM injection model that simultaneously elicits reduced perfusion and soft tissue fibrosis in a hindlimb, in a non-surgical setting. The model allows longitudinal perfusion monitoring (via LDPI) alongside morphological assessment, offering a translationally relevant bridge between models of pure fibrosis and pure ischemia.

Traditional models of BLM-induced skin fibrosis are well-characterized: Repeated intradermal or subcutaneous BLM injections promote dermal thickening, collagen deposition, immune cell infiltration, and α-SMA-positive myofibroblast presence. 5 Yet because it is largely limited to skin, it does not permit functional perfusion assessment in limbs or vascular dysfunction readouts. 8 11

Conversely, murine HLI models—typically via femoral artery ligation or excision—are extensively used in vascular biology, with LDPI serving as a common non-invasive technique to monitor superficial blood flow recovery (measurement depth ≈0.3–1 mm). 12 The Journal of Visualized Experiments protocol for LDPI emphasizes the need to standardize scanning conditions, hair removal, and temperature control for valid measurements. 10 However, LDPI's limitation is that it measures only superficial perfusion; deeper tissue perfusion may remain compromised even when LDPI suggests recovery. 13 In support of this, Becker et al demonstrated that while LDPI suggests restored perfusion during recovery, contrast-enhanced ultrasound (CEUS) reveals that deeper tissue perfusion remains suboptimal. 14 In a comparative ischemia model, Hendrikx et al showed that LDPI significantly underestimates perfusion recovery relative to ^99m^ Tc-sestamibi SPECT (single-photon emission computerized tomography) imaging. 13

Because fibrosis models lack functional perfusion metrics and ischemia models lack a fibrotic microenvironment, our model addresses a gap: the induction of both fibrosis and vascular compromise in a single limb. In our experiments, BLM-treated limbs developed progressive edema, skin induration, and joint stiffening. Serial LDPI showed reduced perfusion that persisted at later time points, consistent with sustained microcirculatory compromise. Histological evaluation showed increased dermal thickness, dense collagen deposition, inflammatory infiltrates, and remodeling of microvessels (narrower lumens, lower vessel density relative to control). These findings indicate that localized BLM injection into a limb compartment can drive microangiopathic remodeling along with fibrotic change.

In this study, vasculopathy was defined operationally by the combination of persistent functional perfusion reduction on LDPI and histologic evidence of microvascular alteration, including reduced CD31-positive signal and vascular remodeling on H&E sections.

Previous studies using BLM-induced skin fibrosis models have suggested overlap between fibrotic and vascular pathways. For example, the Areg (amphiregulin)–epidermal growth factor receptor (EGFR)-mitogen-activated protein kinase kinase (MEK) axis is upregulated during BLM-induced skin fibrosis, and Areg-knockout mice resist BLM-induced fibrosis as previously reported in BLM-induced fibrosis models. 15 Furthermore, studies using BLM skin models have tested antifibrotic agents that also preserve capillaries; for instance, LG283 was shown to reduce dermal thickening and trichrome-positive collagen in BLM skin while maintaining vascular integrity (i.e., preventing capillary loss). 16 Also, mirodenafil was reported to ameliorate skin fibrosis in a BLM-induced SSc mouse model. 17 These observations suggest that dual antifibrotic and vascular-protective strategies may succeed, and our limb model could offer a stricter testbed for such therapies. Recent work using intralesional adipose-derived stem cells in a BLM-induced SSc model also demonstrated attenuation of dermal fibrosis with modulation of angiogenesis-related readouts, supporting the therapeutic relevance of combined anti-fibrotic and vascular endpoints. 18

Nevertheless, the model has limitations. It does not recapitulate systemic autoimmunity or internal organ involvement typical of diffuse SSc (such as lung or renal fibrosis). Early edema/inflammation following BLM injections may cause mechanical compression of microvessels, transiently reducing perfusion; although we observed persistent perfusion deficits at later time points and structural changes in vessels, this confounding effect cannot be entirely ruled out. Also, perfusion was measured only by LDPI, which assesses superficial flux and does not directly measure deeper tissue perfusion; incorporation of other imaging modalities (e.g. CEUS, MRI, micro-CT) would strengthen validation. Finally, we did not assess functional endpoints such as muscle strength, gait, or metabolic markers; inclusion of such measures in future work would increase translational relevance.

Future studies should extend follow-up periods to observe whether perfusion deficits worsen, stabilize, or adapt; incorporate multimodal perfusion imaging; explore molecular mechanisms of endothelial injury, hypoxia signaling, TGF-β/suppressor of mothers against decapentaplegic, extracellular matrix–vascular interactions; and critically, apply candidate proangiogenic or antifibrotic therapies (e.g., stem/progenitor cells, growth factors, exosomes, small molecules) to test the model's utility for translational screening.

### Conclusion

We established a non-surgical, localized BLM hindlimb model that recapitulates the dual pathology of skin fibrosis and microvascular compromise characteristic of SSc-related extremity disease. Taken together, LDPI-confirmed perfusion deficits, histological fibrosis endpoints, and molecular evidence of myofibroblast activation with capillary rarefaction support the validity of this platform. The model is technically straightforward, reproducible, and permits integrated evaluation of perfusion and tissue remodeling within the same animal. Although it does not capture systemic autoimmunity or internal organ involvement, it fills a methodological gap between fibrosis-only dorsal skin models and surgically induced ischemia models. It is, therefore, well-suited for preclinical screening of proangiogenic or antifibrotic therapies relevant to the reconstructive management of SSc-related hand and digit complications.
