# Supplementary material for: Development of Local Bleomycin-Induced Hindlimb Model Recapitulating Skin Fibrosis and Vasculopathy of Systemic Sclerosis
Source: Arch Plast Surg. 2026 Aug 11;53(4):378–85. doi: 10.1055/a-2908-5229 (PMC13496216; doi:10.1055/a-2908-5229)
Supplement: Supplementary file 1 [file 10-1055-a-2908-5229_29351546.pdf]

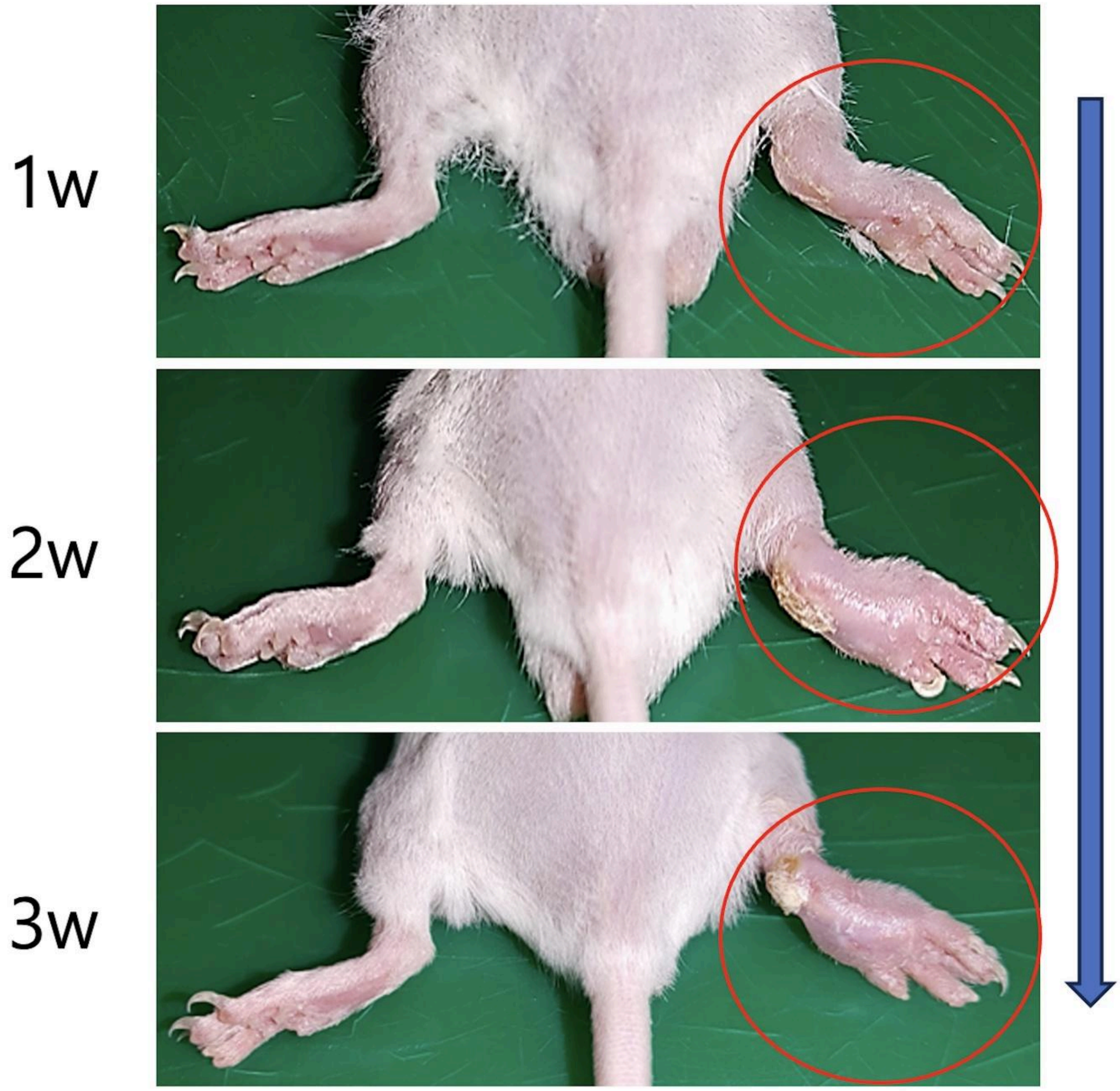


**Supplementary Fig. 1** Time-course of hindlimb changes after 300 μg bleomycin injection: Sequential photographs of the right hindlimb at 1, 2, and 3 weeks. After 2 weeks of daily bleomycin administration, marked edema was observed, and joint contracture became evident at week 3 during the drug-free rest period.
